# Uncovering New Pathogen–Host Protein–Protein Interactions by Pairwise Structure Similarity

**DOI:** 10.1371/journal.pone.0147612

**Published:** 2016-01-22

**Authors:** Tao Cui, Weihui Li, Lei Liu, Qiaoyun Huang, Zheng-Guo He

**Affiliations:** National Key Laboratory of Microbiology, Huazhong Agricultural University, Wuhan, 430070, China; Texas A&M University, UNITED STATES

## Abstract

Pathogens usually evade and manipulate host-immune pathways through pathogen–host protein–protein interactions (PPIs) to avoid being killed by the host immune system. Therefore, uncovering pathogen–host PPIs is critical for determining the mechanisms underlying pathogen infection and survival. In this study, we developed a computational method, which we named pairwise structure similarity (PSS)-PPI, to predict pathogen–host PPIs. First, a high-quality and non-redundant structure–structure interaction (SSI) template library was constructed by exhaustively exploring heteromeric protein complex structures in the PDB database. New interactions were then predicted by searching for PSS with complex structures in the SSI template library. A quantitative score named the PSS score, which integrated structure similarity and residue–residue contact-coverage information, was used to describe the overall similarity of each predicted interaction with the corresponding SSI template. Notably, PSS-PPI yielded experimentally confirmed pathogen–host PPIs of human immunodeficiency virus type 1 (HIV-1) with performance close to that of *in vitro* high-throughput screening approaches. Finally, a pathogen–host PPI network of human pathogen *Mycobacterium tuberculosis*, the causative agent of tuberculosis, was constructed using PSS-PPI and refined using filtration steps based on cellular localization information. Analysis of the resulting network indicated that secreted proteins of the STPK, ESX-1, and PE/PPE family in *M*. *tuberculosis* targeted human proteins involved in immune response and phagocytosis. *M*. *tuberculosis* also targeted host factors known to regulate HIV replication. Taken together, our findings provide insights into the survival mechanisms of *M*. *tuberculosis* in human hosts, as well as co-infection of tuberculosis and HIV. With the rapid pace of three-dimensional protein structure discovery, the SSI template library we constructed and the PSS-PPI method we devised can be used to uncover new pathogen–host PPIs in the future.

## Introduction

Upon infection by pathogens, various host immune response pathways such as toll-like receptors signaling, NF-κB signaling, phagocytes, and cell-apoptosis pathways are activated. These pathways then work collectively to recognize, take up, and ultimately kill invading pathogens. However, pathogens have evolved diverse strategies for survival and replication under hostile host environments [1–3]. Pathogens usually counteract host immune defense and even acquire host nutrition through physical protein–protein interactions (PPIs). For example, the *Legionella* effector RavZ inhibits host autophagy through irreversible Atg8 deconjugation [4]. The VirA protein of *Shigella flexneri* targets host Rab1 for inactivation and then contributes to *Shigella* escape from autophagy [5]. *Neisseria* TbpA binds iron-containing transferrin and extracts iron directly from serum transferrin in a human host [6]. Therefore, discovery and analysis of pathogen–host PPIs can enable the determination of pathogen survival mechanisms in hosts.

Pathogen–host PPIs have been screened for several viruses [7–10] and bacteria [11–14] because they are critical for our understanding of pathogen-survival mechanisms. Information on such interactions are available though several organism-specific or comprehensive-public databases, including the Human Immunodeficiency Virus (HIV)-1 Human-Interaction Database [15], VirusMentha [16], and HPIDB [17]. Experimental approaches mostly used to map pathogen–host PPIs include affinity purification coupled with mass spectrometry [7] and yeast two-hybrid assay [8–11]. However, reliable experiment-based methods are time-consuming, expensive, and applicable only in limited species. Computational methods may play important roles in paving the way for experimental pathogen–host PPI verifications by highlighting high potential interactions and limiting the experimental scope, which can help to reduce expenses and accelerate the pace of discovery [18]. Computational methods for pathogen-host PPIs include the 'interologs' method [19, 20], domain-domain interaction method [20, 21], structure similarity-based method [22, 23] and machine learning-based method [24].

Tuberculosis (TB) causes two million deaths annually worldwide, and approximately one-third of the world’s population is asymptomatically infected with *Mycobacterium tuberculosis*, the main causative agent of this disease. TB infection also activates HIV replication and exacerbates HIV infection [25]. Successful intracellular survival of *M*. *tuberculosis* in macrophages involves modulation of several host-cell processes, including innate immune response and phagosome maturation [26–28]. However, the molecular mechanisms underlying such processes are unclear, which has been a stumbling block for development of efficient therapeutics.

In the present study, we developed a computational method, which we call pairwise structure similarity-PPI (PSS-PPI) to predict pathogen–host PPIs. PSS-PPI is a structure similarity based method with improvement compared to previous methods [22, 23], including more credible templates and a score function that measures similarity of both global structure and local interaction interface. PSS-PPI uses highly credible complex structures from PDB database as templates to predict new PPIs and integrates structure similarity with residue contact information to score the credibility of each new predicted interaction. Results showed that PSS-PPI successfully recovered experimentally confirmed pathogen–host PPIs of human immunodeficiency virus type 1 (HIV-1) with performance close to that of *in vitro* high-throughput screening. We then constructed a pathogen–human PPI network of human pathogen *M*. *tuberculosis* using PSS-PPI. Network-analysis results indicated that the serine/threonine protein kinase (STPK) family, the ESAT-6 secretion system (ESX) family, and the PE/PPE family proteins of *M*. *tuberculosis* interact with human proteins involved in immune response and phagocytosis pathways. Comparison with the HIV-human PPI network indicated that *M*. *tuberculosis* also targets human proteins involved in HIV infection. These findings can serve as a basis for understanding the interaction between *M*. *tuberculosis* and human host at a molecular level and can also provide insights into the molecular mechanisms of co-infection by TB and HIV.

## Materials and Methods

### Construction of SSI template library

The SSI template library was constructed using experimentally resolved, high-quality, three-dimensional complex structures in the PDB database [29]. At the last data update of the current study, the PDB database contained a total of ~100,000 structures. By excluding structures of non-protein polymer (DNA/RNA), as well as monomeric and homomeric proteins, ~17,000 heteromeric protein complex structures were selected. Structures that were not resolved by X-ray diffraction or NMR-based methods were removed. In addition, structures resolved by X-ray diffraction with >4.0 Å resolution were removed. Finally, ~16,000 structures were collected and downloaded from the PDB database. Then, SSIs were identified from these complex structures by calculating inter-chain residue–residue contacts. Each complex structure was first split into monomer chains. Residue–residue contacts between monomer chains were then calculated by measuring atom–atom contacts using 6.05 Å as the cutoff distance [30]. A total of ~40,000 SSIs with >50 residue–residue contact number were identified from ~16,000 complex structures. Given the redundancy of the PDB database, our current collected SSIs were also redundant. Each SSI corresponded to a PPI, and corresponding SSIs of a PPI were usually largely identical with minor differences. At present, structure-similarity alignment is more computationally intensive than sequence-similarity alignment. Therefore, it is necessary to remove redundancy in our SSI template library. In the current study, we used a “max RRCN” strategy to construct a non-redundant SSI template library. That is, if two or more SSIs were complex structures corresponding to the same PPI, only the SSI with the **m**aximal **r**esidue–**r**esidue **c**ontacts **n**umber (max RRCN) was selected as representative SSI. Finally, we obtained a high-confidence and non-redundant SSI template library containing 3,375 SSIs out of 6,267 structures.

### Protein sequences and structures

Proteins sequences of the *M*. *tuberculosis* proteome (3982 proteins) and human proteome (20,272 proteins) were obtained from the UniProt database [31]. The three-dimensional structures of proteins were identified by sequence-similarity alignment using BLAST+ [32]. First, a local BLAST database was constructed using protein sequences of structures in the PDB database [29]. Then, BLAST search for query proteins was performed against the database. Matching structures were required to have >90% sequence identity and >80 amino-acid residues. To minimize redundancy, the matched structures for each protein were grouped according to the corresponding protein segments. The structure with the highest sequence identity in each group was selected as the representative structure. If multiple structures had the same sequence identity, the structure with the best resolution was selected. A total of 443 structures for 423 *M*. *tuberculosis* proteins and 5,859 structures for 4,843 human proteins were obtained. The selected three-dimensional structures were downloaded from the PDB database [29].

### Structure–structure alignment

Structure–structure alignment and similarity scoring was performed using the structural superimposition program TM-align [33].

### PSS-PPIs and PSS score

We defined sA and sB as the structures of query proteins pA and pB, respectively, and tA and tB as the structures of a known SSI complex from the SSI template library. Then, a potential interaction between pA and pB was predicted if sA and sB shared structural similarity with tA and tB, respectively.

We defined the PSS-score, which integrated structure similarity and residue–residue contact coverage information to quantify the overall match of the query structures to the corresponding SSI template. PSS-score was calculated as follows:

PSS-score=SIMA×SIMB×COV (1)

where SIMA is the structure-similarity score between query structure sA with template structure tA. In the current study, structure–structure alignments were performed using TM-align, which used a TM-score (ranging from 0.0 to 1.0) to quantity similarity between the two structures. For each pair of structure–structure alignment, the TM-align output of two TM-score values were normalized by the length (residue number) of the two aligned structures. We defined SIMA as the average values of two TM-scores from the alignment of sA and tA. In the same way, SIMB was defined as the average values of two TM-scores from the alignment of sB and tB. COV is the coverage of residue–residue contacts calculated by

$$\text{COV}=\frac{\text{RRCN}(\text{sA},\text{sB})}{\text{RRCN}(\text{tA},\text{tB})}$$ (2)

where RRCN(tA,tB) is the residue–residue contact number of the SSI template. RRCN(tA,tB) was calculated by measuring atom–atom contacts. A residue–residue contact was defined if the distance of any heavy atom pair from two residues was shorter than the cutoff value of 6.05 Å [30]. RRCN(sA,sB) is the residue–residue contact number of the predicted interaction model. Notably, RRCN(sA,sB) was mapped from residue–residue contacts in SSI template according to residue-alignment information generated by structure alignment.

### Pathogen-human protein–protein interaction data

HIV-1 and human proteins known to regulate HIV-1 replication were obtained from the HIV-1 Human-Interaction Database [15]. Comprehensive pathogen–host protein interaction data sets were obtained from the VirusMentha Database [16].

### Visualization of protein interactions

Protein interaction data was visualized using Cytoscape software [34]. Each protein was represented as a node. Two proteins were linked by an edge if they interacted with each other.

## Results

### Principle of PSS-PPI and PSS score

**Fig 1:**
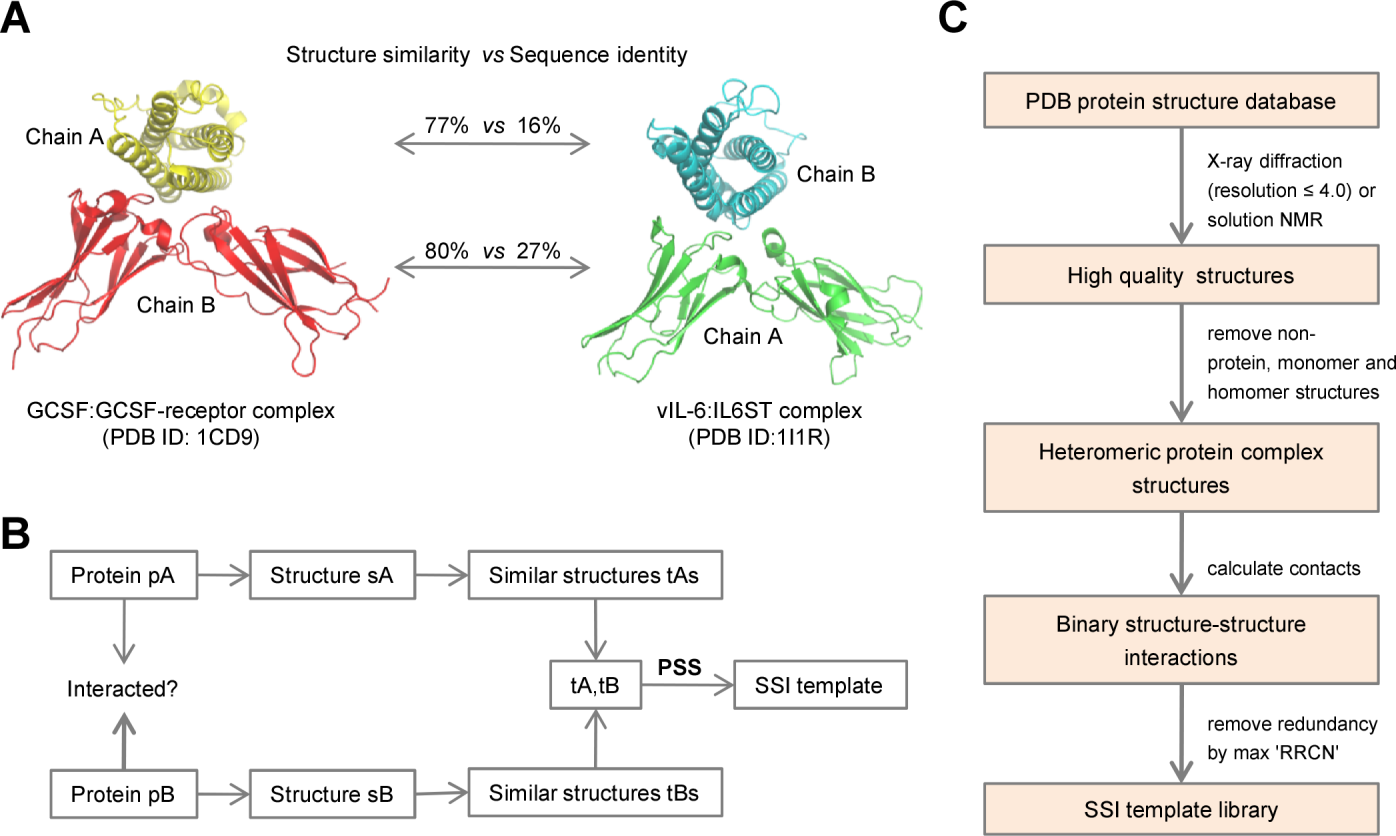
PSS-PPI and SSI template library. (A) An example of PSS. 1CD9 is the complex structure of GCSF (chain A) and GCSF-receptor (chain B). 1I1R is the complex structure of human herpesvirus 8 protein vIL-6 (chain B) and human interleukin-6 receptor beta subunit IL6ST (chain A). 1I1R and 1CD9 are structurally similar but have very low sequence similarity. GCSF means granulocyte colony-stimulating factor. (B) Schematic of PSS-PPI. (C) Flow chart showing the approach used for constructing the SSI template library.

Structurally similar monomer proteins can share similar interactions even in the absence of significant sequence similarity. For example, the GCSF:GCSF-receptor complex structure and vIL-6:IL6ST complex structure share structurally similar monomer and interaction conformations (Fig 1A*)*. Structure and sequence alignment indicated that GCSF with vIL-6 and GCSF-receptor with IL6ST shared 77% and 80% structure similarity, respectively, whereas their sequence identities were only 16% and 27%, respectively. This observation indicated the possibility that known complex structures can be used to discover potential new protein interactions using structure similarity as a bridge. In the current example, interaction between vIL-6 and IL6ST can be inferred based on pairwise structure similarity (PSS) of vIL-6 and IL6ST monomers to monomers in the GCSF:GCSF-receptor complex.

Based on this observation, we proposed a computational method, which we named PSS-PPI, to predict pathogen–host PPIs. PSS-PPI is based on the idea that given two proteins pA and pB with structures sA and sB that shared structural similarity with tA and tB, respectively, from a known tA:tB complex, then pA and pB are also likely to interact with each other (Fig 1B*)*.

The GCSF:GCSF-receptor complex mentioned in the above example was used as a structure–structure interaction (SSI) template. In order to apply PSS-PPI, a large number of such SSI templates were required. Accordingly, we comprehensively explored the experimentally resolved three-dimensional heteromer complex structures in the PDB database [29]. We obtained ~22,000 highly credible binary SSIs. After removing redundant SSIs, the final SSI template library contained more than 3,000 non-redundant SSIs (Fig 1C, Methods).

Interactions with a high degree of similarity between query structures and template structures can be reasonably predicted to be bonafide interactions. Therefore, it is important to establish a method for quantifying the overall similarity of query structures to structures in the SSI template. Scores from structure alignment may provide the necessary information but may be insufficient and may lead to false positives when a similar region is not located in the region that actually mediates the interaction. Therefore, we introduced residue–residue contact coverage to quantify the overlap between structural region sharing similarities with regions that actually mediate interaction. Finally, an integrated score, which we named the PSS score, was calculated by integrating structure-similarity information and residue–residue contact-coverage information to assign a global similarity metric for the newly predicted interaction with the corresponding SSI template (Methods).

### Assessment of PSS-PPI

**Fig 2:**
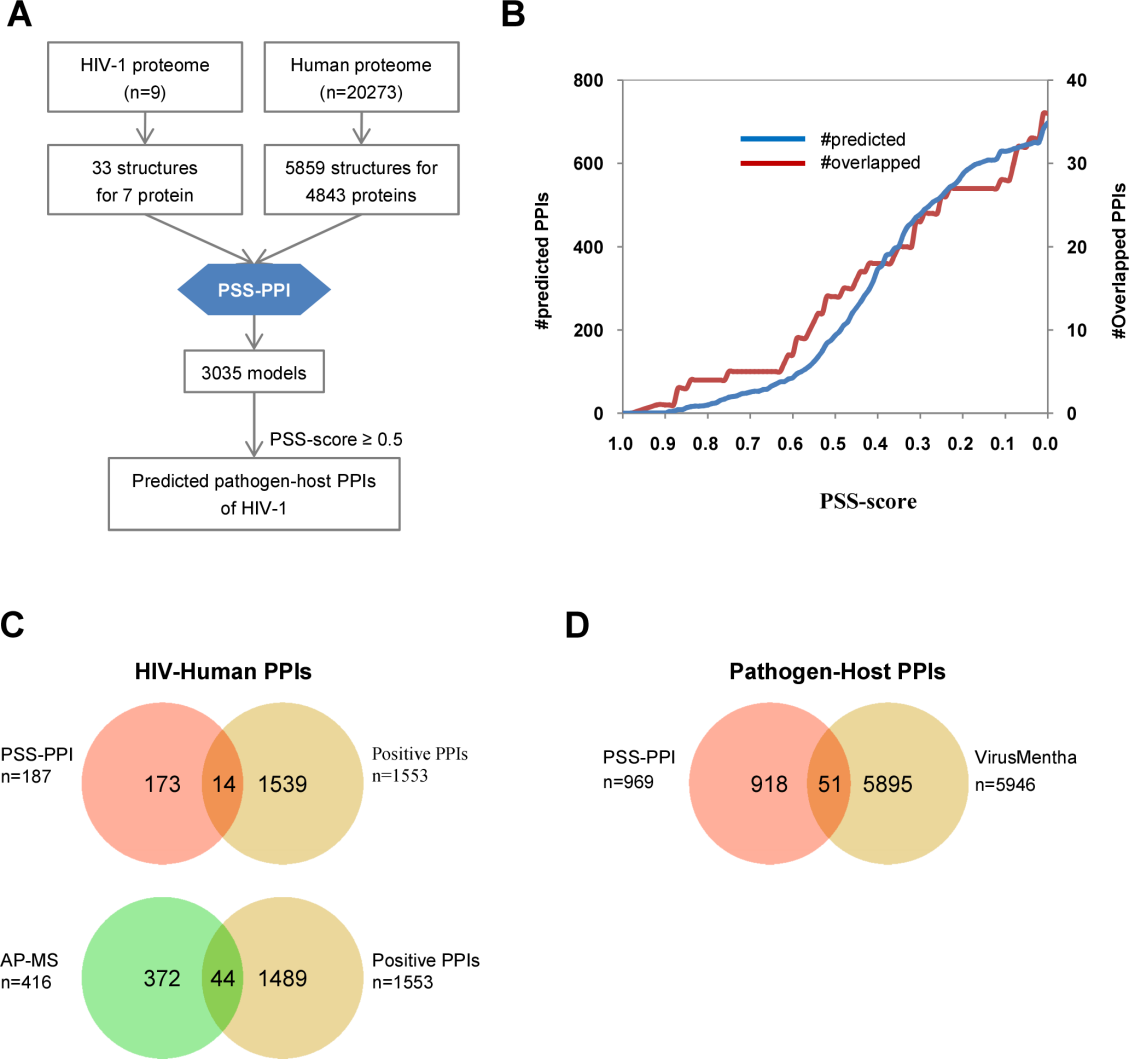
Performance of PSS-PPI. (A) Flow chart showing construction of the pathogen–human PPI network of HIV-1 using PSS-PPI. (B) Predicted PPI number and overlapped positive PPI number vs. PSS score. (C) Venn diagram of overlap between HIV-human PPIs from PSS-PPI (PSS-score cutoff = 0.5), HIV-human protein-interaction database and high-throughput screening.

To examine the capability of PSS-PPI, we applied this method to predict the pathogen–host PPIs of HIV-1 (Fig 2A). The predicted PPIs were compared with the positive dataset that included 1,538 HIV-human PPIs obtained from the HIV-1 Human-Interaction Database [15]. The overlapped PPI number increased with decreased PSS score (Fig 2B). The ratio between overlapped PPIs with predicted PPIs increased with PSS score (S1 Fig), suggesting that higher PSS score implied better prediction. This finding indicated the capability of the PSS-PPI method to discover the pathogen–host PPIs of HIV-1. We further compared the performance of PSS-PPI with high-throughput experimental screening [7]. At a PSS-score cutoff of 0.5, 14 out of the 187 PPIs predicted by PSS-PPI overlapped with positive PPIs. By contrast, 44 out of 416 PPIs generated from high-throughput experiment overlapped with positive PPIs (Fig 2C). Notably, the ratio of overlapped PPIs to predicted PPIs from PSS-PPI (14/187 = 0.075) was close to the ratio of overlapped PPIs to PPIs generated from high-throughput screening (44/416 = 0.106). Three common PPIs were further identified between PSS-PPI and the high-throughput experimental screen, which suggested that PPIs obtained from the two approaches were largely complementary.

To further determine the applicability of the PSS-PPI method for other pathogens, we applied PSS-PPI to predict pathogen–host PPIs in the scope of the VirusMentha Database. This database collects literature-curated, credible pathogen–host PPIs that are mostly derived from small-scale targeted studies [16]. Using 0.5 as a PSS-score cutoff, 51 out of 969 new predicted PPIs overlapped with positive PPIs in the VirusMentha Database (Fig 2D). These results demonstrate the capability of the PSS-PPI method to discover pathogen–host PPIs.

### Predicting pathogen–host protein interactions of *M*. *tuberculosis*

**Fig 3:**
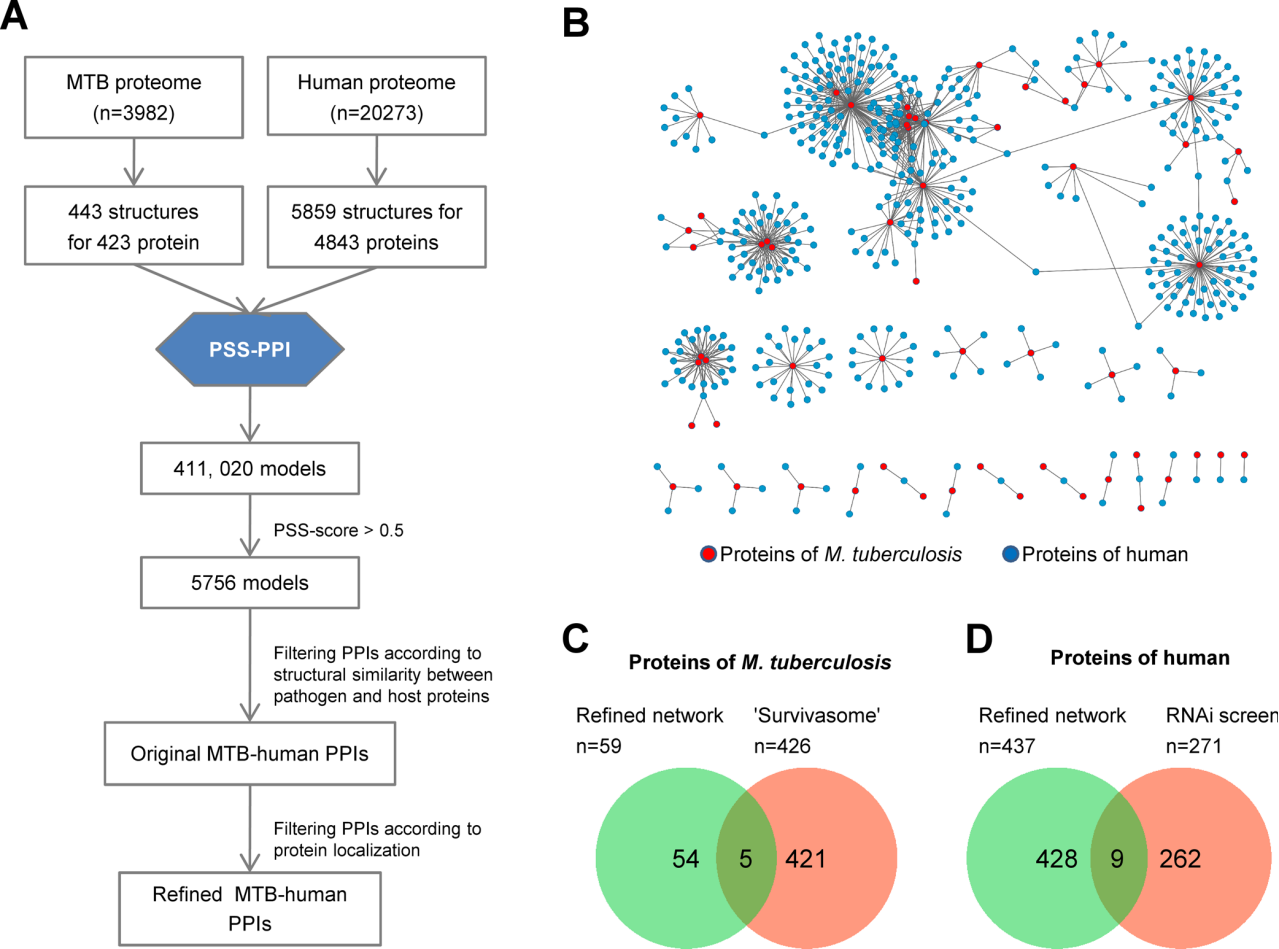
Pathogen–host PPI network of *M*. *tuberculosis*. (A) Flow chart showing the construction of pathogen–host PPI network of *M*. *tuberculosis* using PSS-PPI. (B) Graphical representation of predicted pathogen–host PPI network of *M*. *tuberculosis*. Nodes represent proteins of *M*. *tuberculosis* (red) and human (blue). Edges represent predicted interactions. (C) Overlap between *M*. *tuberculosis* proteins in predicted PPI network and “survivasome” of *M*. *tuberculosis*. (D) Overlap between human proteins in the predicted PPI network and human proteins involved in *M*. *tuberculosis* infection identified by RNAi screening.

We used PSS-PPI to predict pathogen–host PPIs of the human pathogen *M*. *tuberculosis* (Fig 3A). First, protein sequences of *M*. *tuberculosis* and the human proteome were downloaded from the UniProt Database. The representative structures for each protein were assigned by sequence-similarity alignment using BLAST search. A total of 443 structures for 423 *M*. *tuberculosis* proteins and 5,859 structures for 4,843 human proteins were obtained. Using TM-align, all-against-all structure alignment was performed between all 6,302 query protein structures (from *M*. *tuberculosis* and human) and 8,776 structures in the SSI template library. Structures with TM score ≥0.5 with each query structure were deemed similar [33]. Then, 2,595,537 structure–structure pairs between *M*. *tuberculosis* and human were used to query the SSI template library to identify PSS, which led to the identification of 411,020 matching models. The PSS score for each model was calculated and assigned to the corresponding PPI. If more than one model corresponded to a PPI, the maximal score was used. To reduce false-positive interactions, we used two filtration steps to further refine our predicted *M*. *tuberculosis*-human PPIs. The first filter removed PPIs whose structure similarity between *M*. *tuberculosis* protein and human protein were greater than 0.6. This filter was based on our analysis of structure similarity between pathogen and host protein of known pathogen-host PPIs. More specifically, we calculated the structure similarity between pathogen protein and host protein of each known pathogen–host PPI from the VirusMentha database. Then, we analyzed the distribution of these structure similarity scores. The result indicated that structure similarity between pathogen and host protein of almost all known pathogen–host PPIs (1538/1541 = 99.81%) were less than 0.6 (S2A Fig). This suggested that potential PPIs whose pathogen protein and host protein have structural similarity score greater than 0.6 were likely to be false-positive PPIs. However, more than one-third of the predicted PPIs had structure similarity score between *M*. *tuberculosis* protein and human protein greater than 0.6 in our predicted *M*. *tuberculosis*-host PPIs (S2B Fig). Therefore, PPIs with structure similarity score between *M*. *tuberculosis* protein and human protein greater than 0.6 were removed to reduce false-positive predictions.

The second filter removed PPIs according to protein-localization information. Unlike viruses, bacteria have compartmentalized cell structure. This structure should be considered when assessing the feasibility of interactions under *in vivo* conditions in order to decrease false positives. Proteins from the secretome of pathogenic bacteria are more likely to interact with a host. We manually curated culture supernatant proteins and exported proteins of *M*. *tuberculosis* to build the secretome of *M*. *tuberculosis* using data from several publications [35–37]. Subsequently, PPIs in which the pathogen protein was not found in our constructed secretome were removed.

After these two filtration steps, we finally obtained 773 predicted PPIs between 59 *M*. *tuberculosis* proteins and 437 human proteins using 0.5 as the PSS score cutoff (Fig 3B and S1 Table). The PSS score distribution of all PPIs can be found in S3 Fig. The degree of a protein in the PPI network refers to the number of its interacting partners. The degree distribution of proteins in our predicted PPI network approximately followed a power law function (S4 Fig). This suggested that the network was scale-free, which is a general characteristic of biological networks [38].

We manually curated proteins involved in the infection and survival of *M*. *tuberculosis* in a host to build the “survivasome” of *M*. *tuberculosis* from several transposon screening studies [39–41]. We found that 5 out of 59 *M*. *tuberculosis* proteins in our network overlapped with the “survivasome” of *M*. *tuberculosis* (Fig 3C). In addition, we collected host factors responsible for *M*. *tuberculosis* infection from genome-wide RNAi screening studies [42]. We obtained 275 proteins that overlapped with 9 out of 437 human proteins in our network (Fig 3D).

To examine the host-cell process targeted by *M*. *tuberculosis*, we analyzed human proteins in the *M*. *tuberculosis*–human PPI network in the context of GO terms [43]. We counted the frequency of involved GO biological processes (S2 Table). The top groups also included proteins involved in small GTPase-mediated signal transduction, protein transport, innate immune response, and apoptotic process. A considerable number of proteins were also involved in the regulation of signaling pathways linked to NF-κB and phagocytosis.

### *M*. *tuberculosis* targeted host immune-response pathways

**Fig 4:**
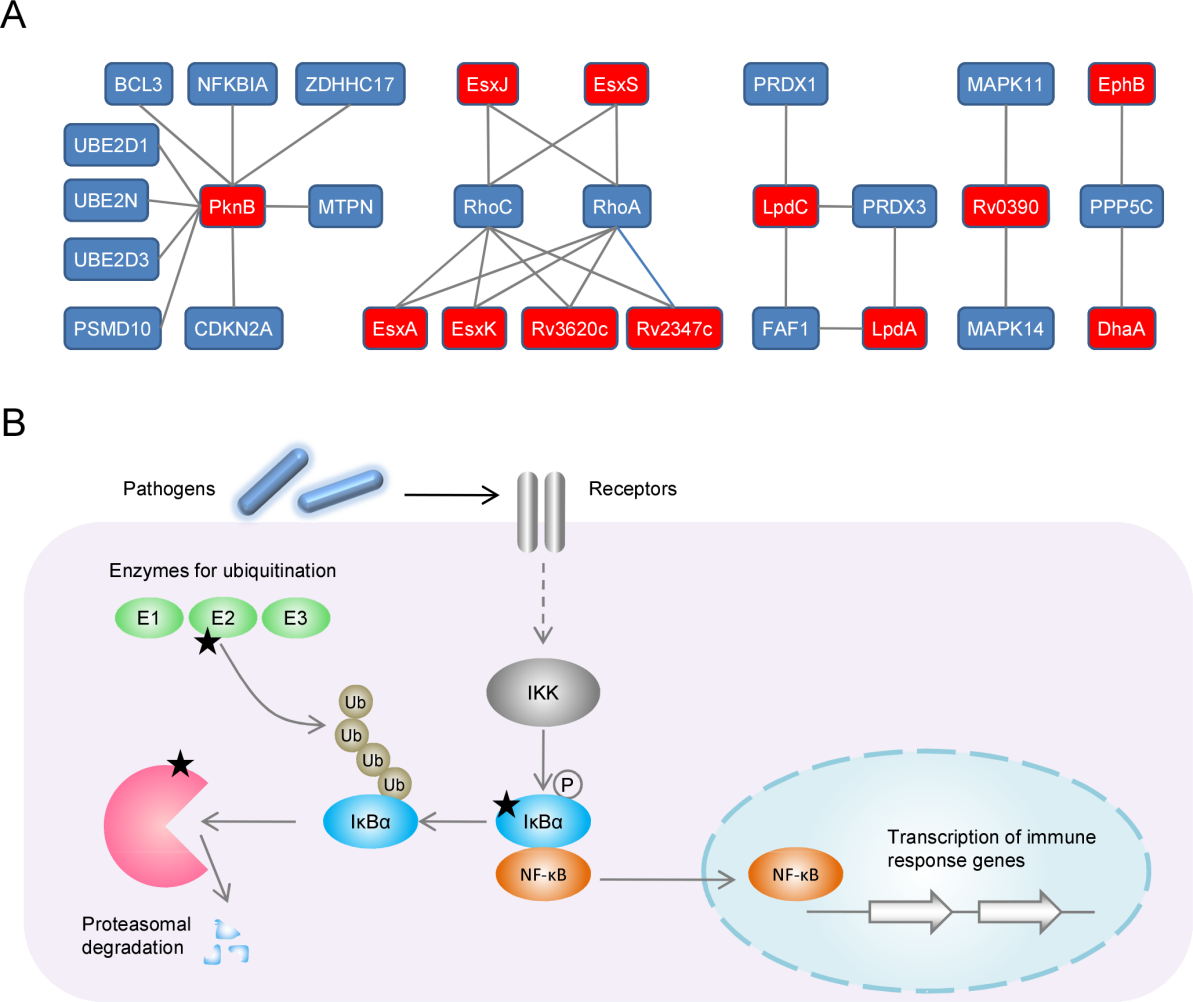
Predicted PPIs between *M*. *tuberculosis* and human immune response pathways. (A) Local PPIs network between *M*. *tuberculosis* proteins (red nodes) and human proteins (blue nodes) involved in the NF-κB signaling pathway. (B) Schematic of the NF-κB signaling pathway. Proteins found to be targeted by *M*. *tuberculosis* are indicated by black stars.

To analyze the interaction between *M*. *tuberculosis* and human anti-microbial pathways, we collected human proteins involved in the NF-κB signaling pathway according to GO annotation and constructed a subnetwork involved in these processes (Fig 4A). The highest connected node in this local network was the PknB protein of *M*. *tuberculosis*, which interacted with nine host proteins. The largest group of *M*. *tuberculosis* proteins came from the ESX family, including five ESX-family proteins (EsxB, EsxJ EsxK, EsxP, and EsxW). Host proteins included the inhibitor of κB (IκB) family proteins IκBα and BCL-3, ubiquitin-conjugating enzyme E2, non-ATPase regulatory subunit 10 of 26S proteasome, and transforming proteins RhoA and RhoC. To intuitively describe the roles of these target proteins, a schematic of the NF-κB signaling pathway is shown (Fig 4B). IκBα is the inhibitor of NF-κB, whereas ubiquitin-conjugating enzyme and proteasome are essential components of the ubiquitin–proteasome pathway, which is required for degrading IκBα and activating NF-κB [44, 45]. These results suggested that *M*. *tuberculosis* STPK and ESX-family proteins targeted multiple host regulators involved in the activation of NF-κB pathways.

### ESX-family proteins targeted host proteins involved in phagocytosis

**Fig 5:**
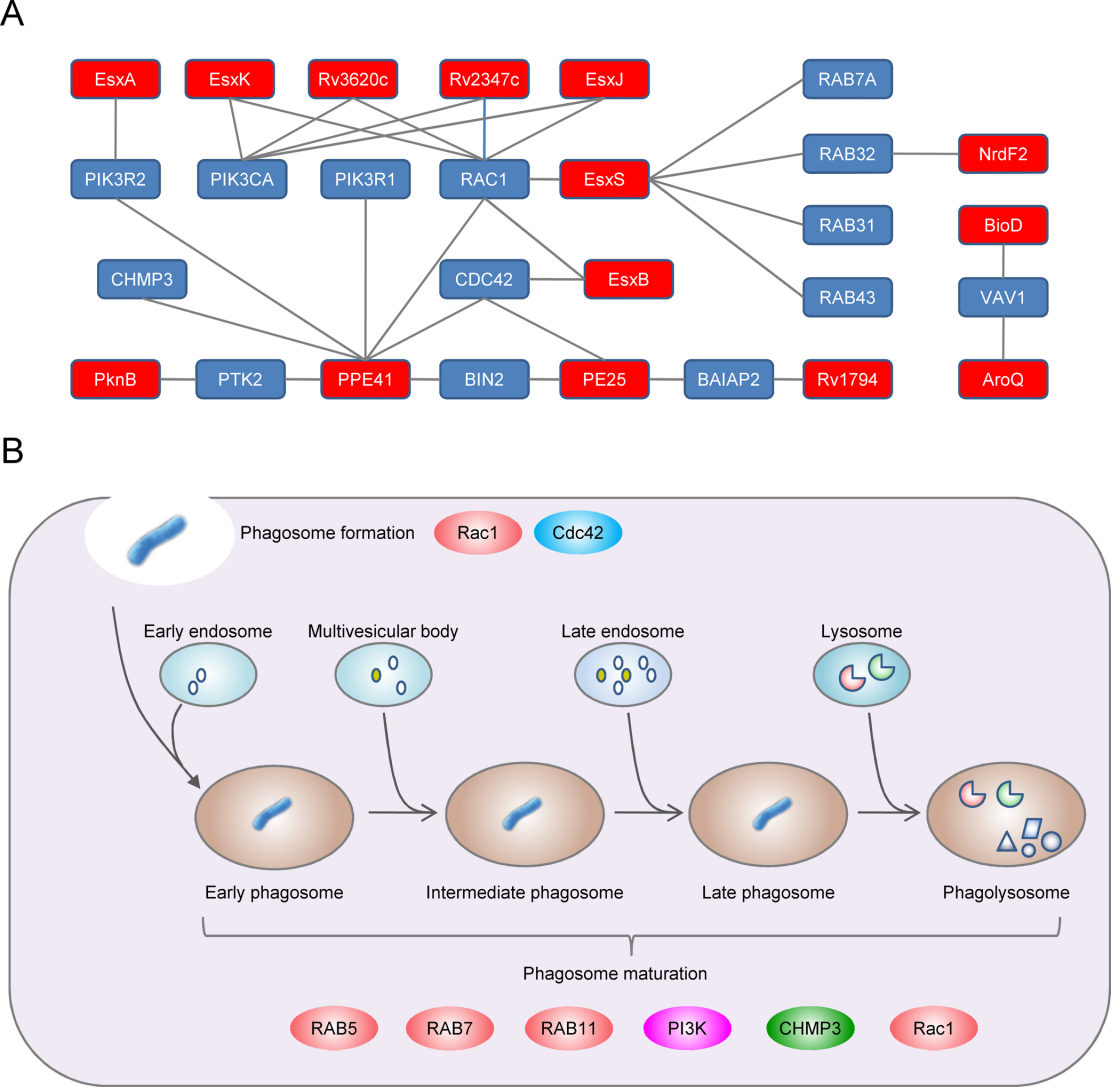
*M*. *tuberculosis* targets human phagocytosis pathways. (A) Local PPI network between *M*. *tuberculosis* proteins (red nodes) and human proteins (blue nodes) involved in phagocytosis. (B) Schematic showing phagocytosis pathways. The RHO family GTPases RAC1 and CDC42 play roles in phagosome formation. Phagosomes undergo sequential fusion with early endosomes, late endosomes and lysosomes. The small GTPase RAB5A is involved in the fusion of phagosomes with early endosomes. The small GTPase RAB7A is known to mediate trafficking between phagosomes and late endosomes or lysosomes. The GTPase RAB11A mediates recycling of endosomes to the plasma membrane. PI3K (PIK3R1, PIK3R2 and PIK3CA) regulates metabolism of phosphoinositides, which play essential roles during phagocytosis. CHMP3 is a component of the ESCRT complex. RAC1 is also involved in stimulation of NADPH oxidase activity in macrophages.

*M*. *tuberculosis* can escape from phagocytosis-mediated antimicrobial activity and survive in a host [26, 46, 47]. To explore the interaction between *M*. *tuberculosis* and the host phagocytosis pathway, we collected host proteins involved in phagocytosis according to GO annotation and predicted interactions that targeted these proteins (Fig 5A). This *M*. *tuberculosis*–human PPI network involved in phagocytosis included 55 interactions between 12 *M*. *tuberculosis* proteins and 20 human proteins. *M*. *tuberculosis* proteins included six ESX-family proteins (EsxA/Rv3875, EsxB/Rv3874, EsxJ/Rv1038c, EsxK/Rv1197, EsxW/Rv3620c, and EsxP/Rv2347c), two PE/PPE family proteins (PE25 and PPE41), and one hypothetical protein Rv1794. Clearly, ESX-family proteins accounted for almost half of *M*. *tuberculosis* proteins that targeted host phagocytosis pathways. Host proteins included multiple small GTPases (RAB5, RAB7, and RAB11), several subunits of phosphatidylinositol 3-kinase PI3K (PIK3R1/P85A, PIK3R2/P85B, and PK3CA), and a component of the ESCRT (endosomal sorting required for transport) complex (CHMP3). To intuitively describe the roles of these target proteins, a schematic of the host phagocytosis-signaling pathway is shown (Fig 5B). Functions of human proteins indicated that *M*. *tuberculosis* may interfere with phagocytosis pathways at multiple stages, including phagosome formation (Rac1 and Cdc42), phagosome maturation (small GTPases RAB5, RAB7, and RAB11, as well as PI3K and CHMP3), and regulation of NADPH oxidase enzyme activity (Rac1) (Fig 5B).

### *M*. *tuberculosis* targeted proteins involved in HIV infection

**Fig 6:**
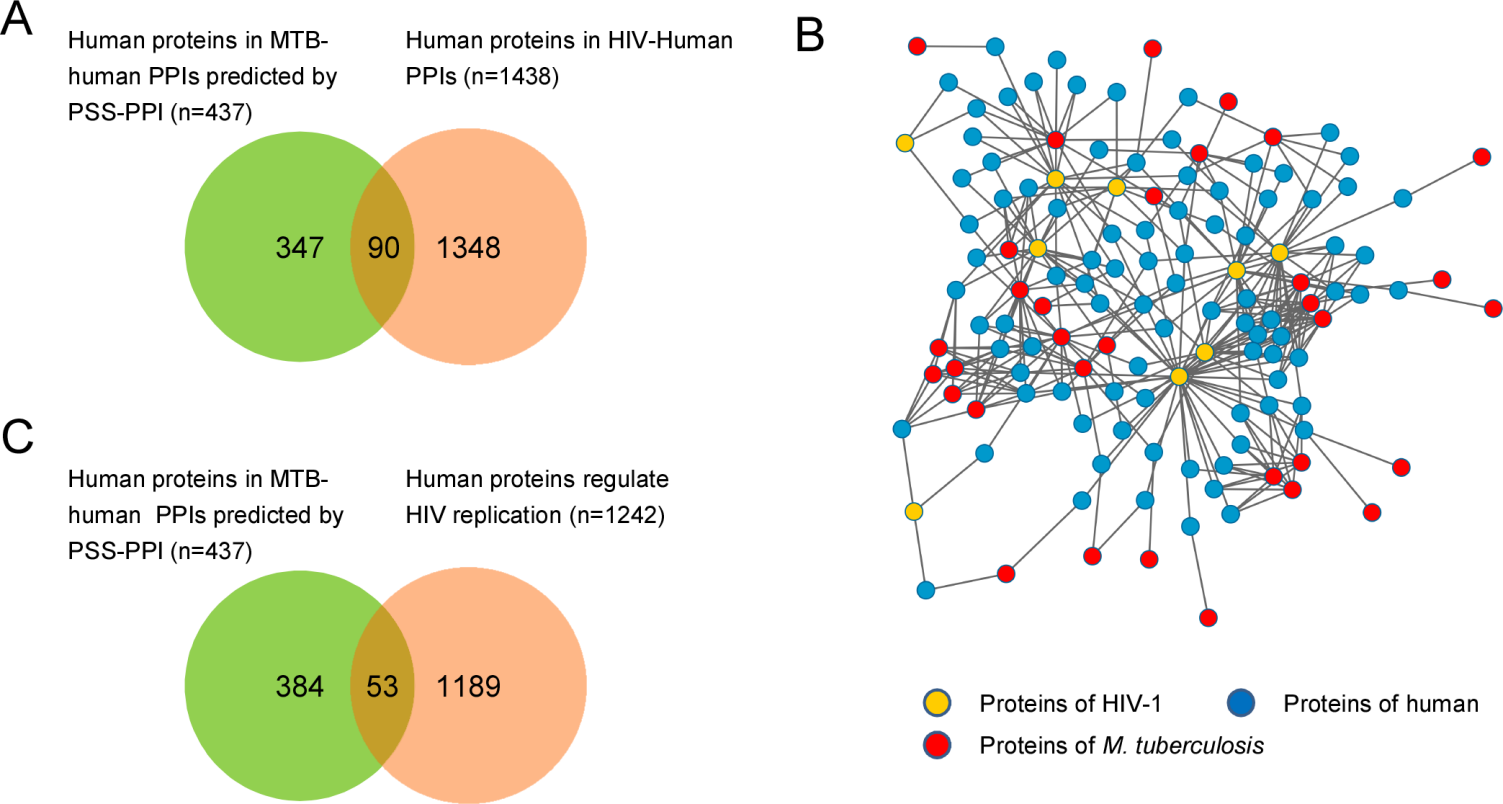
*M*. *tuberculosis* targets human proteins involved in HIV infection. (A) *M*. *tuberculosis* was predicted to target human proteins that are also targeted by HIV-1. (B) The *M*. *tuberculosis*-HIV-human PPI network. Nodes represent proteins of *M*. *tuberculosis* (red), HIV-1 (yellow), and human (blue). (C) *M*. *tuberculosis* was predicted to target human proteins that regulate HIV replication.

HIV infection increases the risk of latent TB reactivation by 20-fold. TB infections activate HIV replication and exacerbate the progression of HIV infection [48, 49]. Exploring the molecular mechanisms underlying these effects can facilitate control of both pathogens. Accordingly, we compared *M*. *tuberculosis*-human PPIs with HIV-human PPIs. We constructed a HIV-human PPI network using PPI data from the NCBI HIV-1 Human-Interaction Database [15]. Interestingly, 90 human proteins in the *M*. *tuberculosis*–human PPI network overlapped with human proteins in the HIV-human PPI network (Fig 6A and 6B). We also found that *M*. *tuberculosis* targeted 53 human proteins involved in regulating HIV replication (Fig 6C). These results suggest that *M*. *tuberculosis* is likely to affect the function of proteins targeted directly by HIV, as well as those that regulate HIV replication.

## Discussion

Discovery of new pathogen–host PPIs is essential for understanding the infection and survival mechanisms of pathogens in a host. In this study, we constructed a comprehensive and non-redundant SSI template library and developed PSS-PPI to uncover new pathogen–host PPIs. Using an HIV-human model system, we demonstrated that PSS-PPI successfully predicted pathogen–host PPIs with performance close to that of *in vitro* experimental high-throughput screening. In addition, PSS-PPI predicted PPIs that were complementary to those predicted by experiment-based screening. Given the scarcity of pathogen–host PPI data for most pathogens, PSS-PPI shows promise for opening new doors to explore the survival mechanism of these pathogens.

All interaction templates known to date have originated mainly from model organisms such as human, yeast, and *Escherichia coli*. Therefore, search for target proteins similar to unique pathogen proteins that are associated with virulence in the interaction template-library have usually failed. This weakness can be overcome by utilizing structural similarity information. PSS-PPI is a structure-based PPI prediction method that uses structural similarity between proteins as a bridge to identify new interactions. Compared with sequence similarity-based methods, this structure similarity-based approach enabled us to explore new interactions of proteins that lacked significant sequence similarity with a known interaction-template library. This advantage is of vital importance in predicting pathogen–host PPIs. Indeed, PSS-PPI enabled the identification of potential interactions of unique ESX and PE/PPE family proteins from *Mycobacteria* in the current study.

PSS-PPI can be classified into template-based PPI prediction methods that transform known interactions to predict new interactions. Therefore, high quality of the interaction-template library (source interactions), including coverage and credibility, is essential for successful application of template-based protein-interaction prediction. In the current study, the SSI template library was constructed with these goals in mind. First, PSS-PPI used templates generated only from known complex structures and was thus more reliable than templates obtained from high-throughput screening. Second, to improve coverage, all currently available structures in the PDB database were considered for identifying SSI templates. In addition, to control quality, complex structures and SSI templates were strictly filtered according to experimental methods, resolution, structure length, and residue–residue contact information.

PSS-PPI used precise structure–structure alignment to define structure similarity. Compared with sequence alignment, structure alignment is computationally intensive. Prediction of new interactions requires the alignment of query proteins to all proteins in the template library. Therefore, the size of the template library is closely associated with computational cost. In this study, we used a “max RRCN” strategy for the first time to reduce template-library redundancy. This strategy reduced the size of the template library while preserving its comprehensiveness and diversity. This optimized template library then enabled practical application of PSS-PPI.

Taking advantage of precise structure–structure alignment information, PSS-PPI predicted new interactions and provided a potential three-dimensional complex model for each predicted interaction. Each potential complex model was generated by superposing two query structures to the corresponding complex template. Despite being rough, this predicted complex model could provide useful information regarding the interaction interface, which was helpful for us to understand interaction mechanisms at residue level and provide information for structure-based inhibitor design.

Structure is the only required input element of PSS-PPI. Therefore, PSS-PPI can be used to predict the pathogen–host PPIs of many other pathogens for which structures are available. Humans are the most important host in pathogen–host studies. A considerable number of human protein structures are available at present, but availability of structural information for pathogen proteins differs greatly. Therefore, utility of PSS-PPI is limited when structure information is incomplete or absent. This obstacle can be overcome by quickly expanding structural information in the PDB database. In addition, structures from homologous modeling can be considered in the future. Nevertheless, our results indicate that the PSS-PPI method can be used to explore the pathogen–host PPIs of pathogens with available structures.

*M*. *tuberculosis* is an obligate intracellular pathogen that can infect and survive in a host. The prediction and analysis of pathogen–host PPIs of *M*. *tuberculosis* is valuable in exploring the survival mechanisms of *M*. *tuberculosis* in a host. In the present study, we predicted the pathogen–host PPIs of the human pathogen *M*. *tuberculosis* using PSS-PPI. This network can serve as a basis for exploring the survival mechanism of *M*. *tuberculosis* within a host.

We found that multiple host factors involved in NF-κB signaling pathways are targeted by *M*. *tuberculosis*. NF-κB family proteins are transcriptional factors that regulate the expression of immune-response genes. IκBα protein inactivates the NF-κB transcription factor by masking the nuclear localization signals of NF-κB proteins and keeping them sequestered in an inactive state in the cytoplasm. Upon cellular stimulation by immune and pro-inflammatory responses, IκB kinase (IKK) specifically phosphorylates IκBα protein. This phosphorylation results in IκBα ubiquitination and degradation. The dissociation of IκBα from NF-κB enables NF-κB to translocate to the nucleus and activate the transcription of immune-response genes [50]. IKK-mediated phosphorylation of IκBα proteins represents a convergence point for most signal-transduction pathways leading to NF-κB activation [51]. In the present study, we identified a potential interaction between Ser/Thr protein kinase PknB and IκBα. This interaction may affect the phosphorylation of IκBα and thereby perturb the activation of NF-κB signaling. Moreover, IκBα degradation requires the ubiquitin–proteasome pathway. Several subunits of the host ubiquitin-conjugating enzyme E2 and proteasome were also found to be targeted by *M*. *tuberculosis* (Fig 4A). Interestingly, PknB has been identified by mass spectrometry in a culture filtrate of *M*. *tuberculosis* H37Rv [37] and *M*. *tuberculosis* H37Rv-infected guinea-pig lungs [52]. This supports the validity of the predicted interactions under *in vivo* conditions.

The pathogenicity of *M*. *tuberculosis* is attributed largely to its ability to survive within macrophages [26, 46, 47]. Interestingly, we found that groups of human proteins involved in phagocytosis were targeted by the ESX-family proteins of *M*. *tuberculosis*. These interactions highlight the essential roles of ESX-family proteins in the pathogen–host interaction of *M*. *tuberculosis*, which is highly consistent with the findings of previous genetic-screening studies [53, 54]. Some of the predicted *M*. *tuberculosis* target proteins such as PI3K and CHMP3 were particularly interesting. PI3K activity is essential for proper phagosomal maturation, and *Mycobacteria* have been shown to use Man-LAN to interfere with PI3K signaling pathways [26, 46]. CHMP3 is a component of the ESCRT complex, and ESCRT has been shown to play roles in restricting the growth of *M*. *tuberculosis* and to be targeted by another ESX family protein EsxH [54, 55]. Therefore, these new predicted interactions suggest an alternative strategy used by *M*. *tuberculosis* to interfere with PI3K signaling pathways and phagosomal maturation. In addition, we found that *M*. *tuberculosis* targeted Rac1, which is involved in activating NADPH oxidase. NADPH oxidase mediates the phagocytic killing of ingested pathogens by producing reactive oxygen species (ROS). Therefore, this interaction may contribute to the observation that *M*. *tuberculosis* is relatively resistant to the microbicidal effects of ROS [56, 57]. Taken together, our results indicate that these interactions are likely to interfere with phagosomal maturation and ROS signaling and are of interest for further experimental exploration.

TB and HIV co-infection is a major challenge in the global control of TB [25]. As mentioned above, HIV infection increases the risk of latent TB reactivation 20-fold. TB infection activates HIV replication and exacerbates the progression of HIV infection [48, 49]. Interactions of HIV and *M*. *tuberculosis* with the human immune system have already been explored at the cellular level [48, 49]. However, their interaction at the molecular level is largely unknown. Interestingly, we found that *M*. *tuberculosis* targets host proteins that regulate HIV replication. We speculate that these interactions may positively regulate the function of host proteins required for HIV replication or negatively regulate the function of host proteins limiting HIV replication. In addition, a group of common host proteins targeted by both *M*. *tuberculosis* and HIV was identified by comparing the *M*. *tuberculosis* PPI network constructed here with known HIV-human protein interactions. We speculate that co-targets of *M*. *tuberculosis* and HIV enhance the interference effect on host cell pathways and facilitate the infection of both pathogens. These findings suggest a concerted attack by *M*. *tuberculosis* and HIV on the host immune system that may contribute to TB-HIV co-infection.

## Conclusions

We constructed an optimized SSI template library and developed a structure-based computational method named PSS-PPI to predict pathogen–host PPIs. We demonstrate that PSS-PPI can effectively discover pathogen–host PPIs with performance close to that of high-throughput screening. Specifically, we constructed a pathogen–host PPI network of the human pathogen *M*. *tuberculosis* using PSS-PPI. Analysis of the network indicated that *M*. *tuberculosis* targeted host immune response and phagocytosis pathways. In addition, *M*. *tuberculosis* targeted host proteins that interact with HIV proteins as well as those that regulate HIV replication. These PPIs provide a resource for exploring the survival mechanisms of *M*. *tuberculosis* in a host and TB-HIV co-infection. With continuous increase in the availability of three-dimensional protein structures in public databases, the SSI template library and the PSS-PPI method reported here can be used to predict pathogen–host PPIs at a larger scale and for diverse pathogens.

## Supporting Information

S1 Fig Ratio of overlapped PPIs and predicted PPIs. The ratio between overlapped PPIs and predicted PPIs increased along with increase in PSS score, suggesting that higher PSS score implied better prediction.
(TIF)

S2 Fig Distribution of structure similarity scores between pathogen protein and host protein in pathogen-host PPIs. (A) PPIs of known pathogen-host PPIs from public databases. (B) PPIs predicted using the PSS-PPI method in the current study.
(TIF)

S3 Fig PSS score distribution of predicted *M*. *tuberculosis*-human PPIs. (TIF)

S4 Fig Degree distribution of the *M*. *tuberculosis*-human PPI network. The degree distribution approximately followed a power law function (y = ax^b^, a = 87.975, b = -1.247, R^2^ = 0.774). This suggested that the predicted PPI network was a scale-free network.
(TIF)

S1 Table *M*. *tuberculosis*-human PPI network predicted by the PSS-PPI method. (XLS)

S2 Table GO term count of human proteins in predicted pathogen-host PPIs of *M*. *tuberculosis*. (XLS)
