# Supplementary figures and images for: Uncovering New Pathogen–Host Protein–Protein Interactions by Pairwise Structure Similarity
Source: PLoS One. 2016 Jan 22;11(1):e0147612. doi: 10.1371/journal.pone.0147612 (PMC4723085; doi:10.1371/journal.pone.0147612)

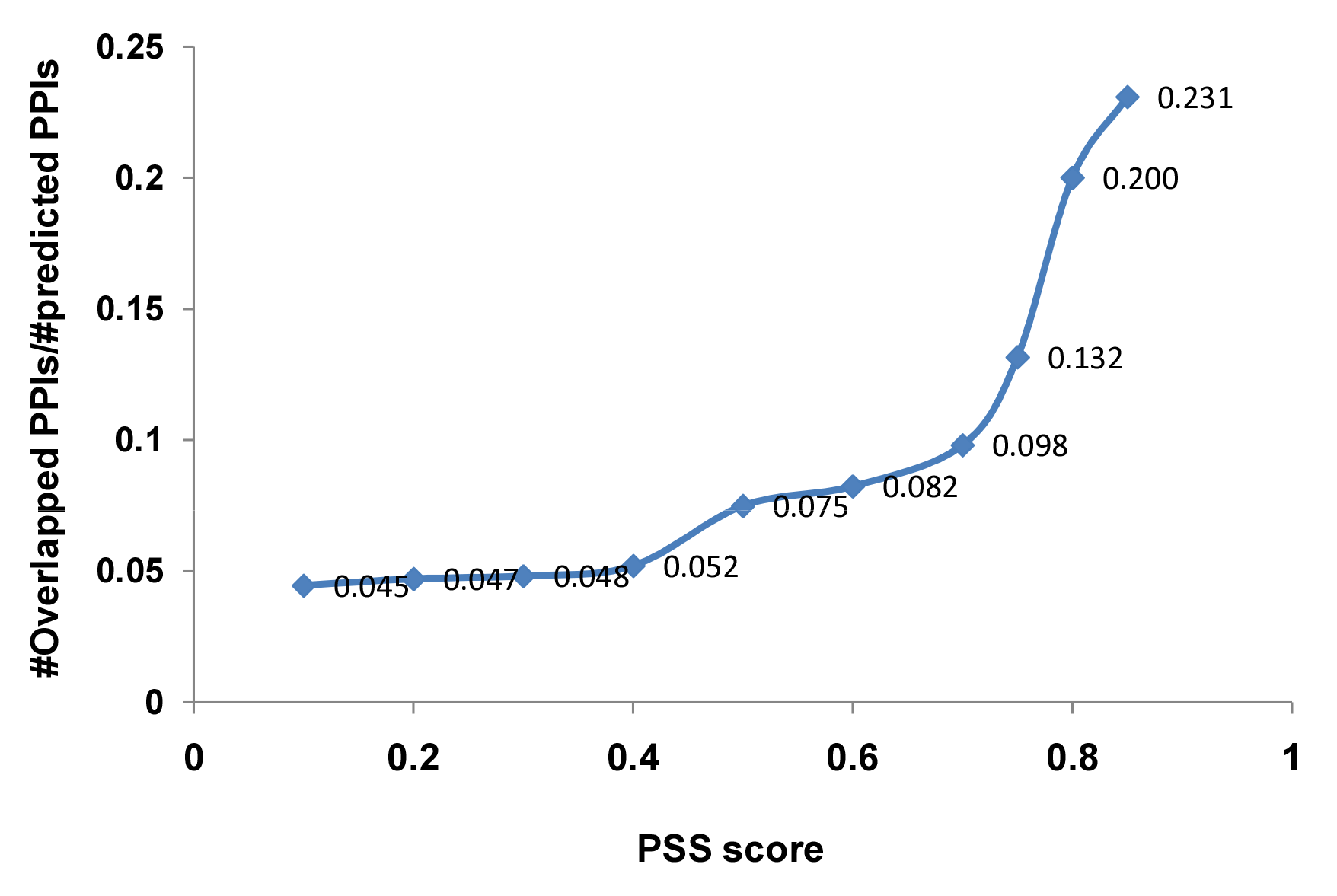

Supplement: S1 Fig — The ratio between overlapped PPIs and predicted PPIs increased along with increase in PSS score, suggesting that higher PSS score implied better prediction. (TIF) [file pone.0147612.s001.tif]

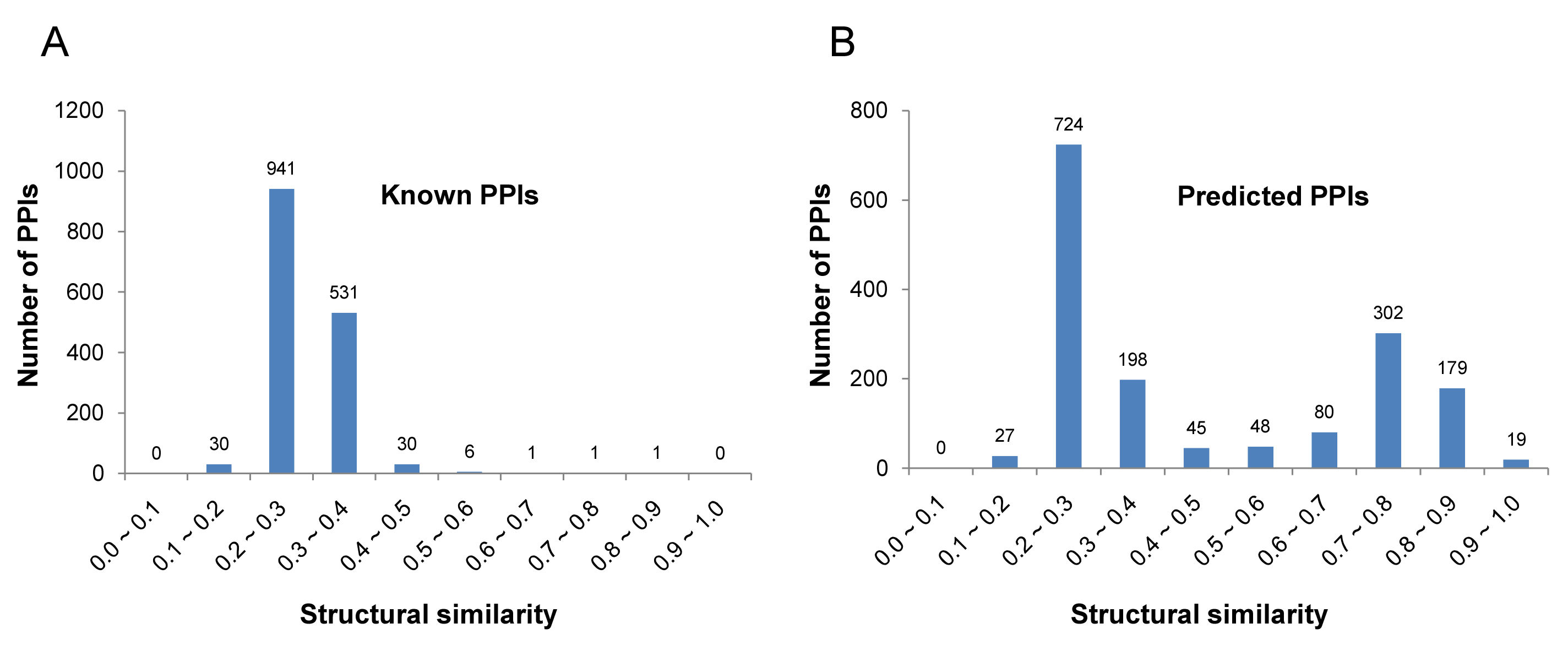

Supplement: S2 Fig — (A) PPIs of known pathogen-host PPIs from public databases. (B) PPIs predicted using the PSS-PPI method in the current study. (TIF) [file pone.0147612.s002.tif]

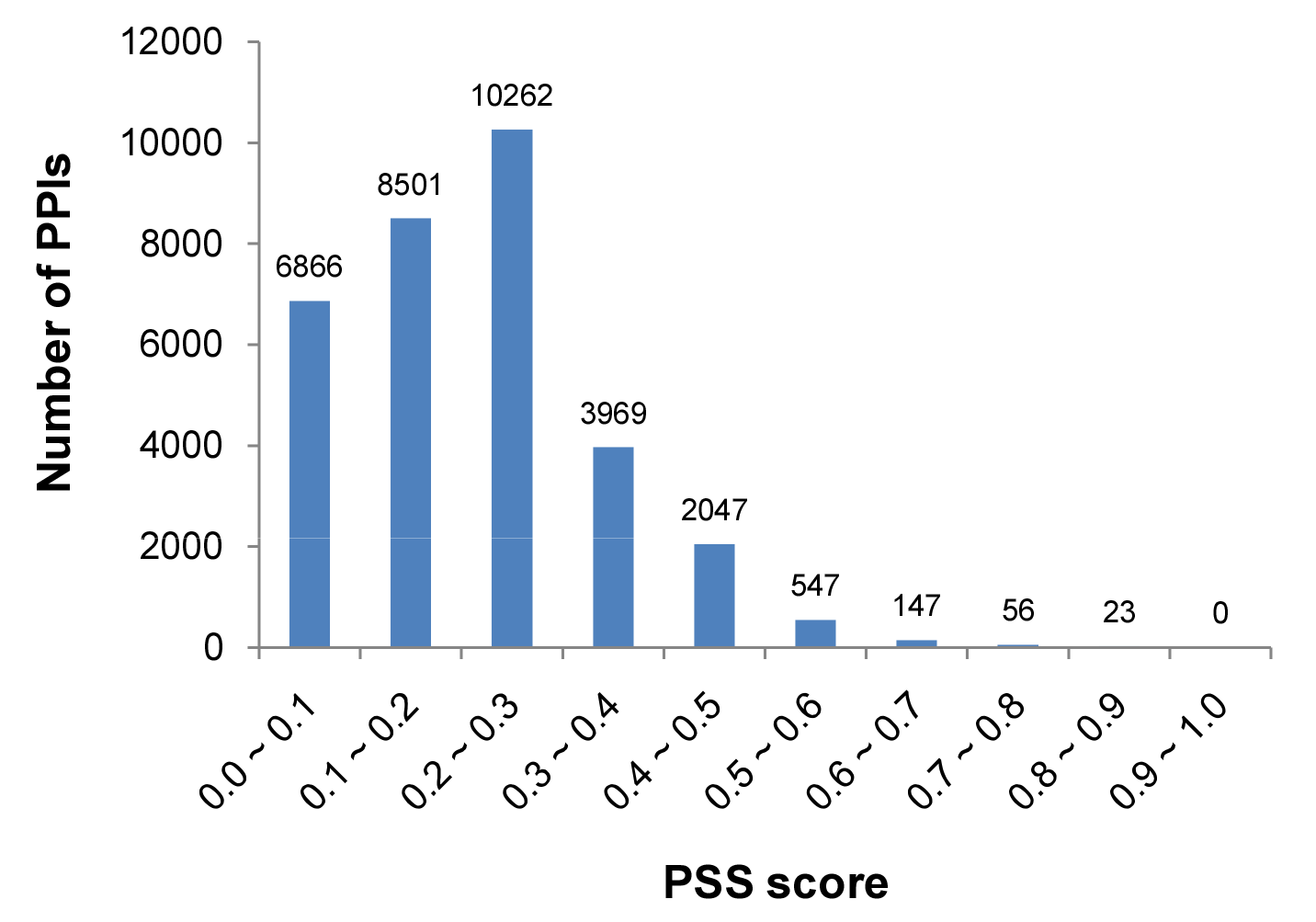

Supplement: S3 Fig — (TIF) [file pone.0147612.s003.tif]

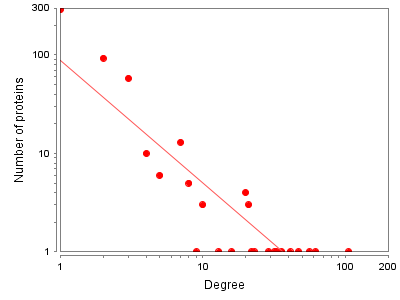

Supplement: S4 Fig — The degree distribution approximately followed a power law function (y = axb, a = 87.975, b = -1.247, R2 = 0.774). This suggested that the predicted PPI network was a scale-free network. (TIF) [file pone.0147612.s004.tif]
